# Flumazenilperfusor bei subakuter Bromazepamintoxikation bei chronischem Abusus

**DOI:** 10.1007/s00101-025-01523-8

**Published:** 2025-03-05

**Authors:** Philippe Conter, Antonia Fritz

**Affiliations:** Klinik für Anaesthesiologie, LMU Klinikum, Marchioninistr. 15, 81377 München, Deutschland

## Anamnese

Wir berichten über eine 74-jährige, adipöse Patientin, die sich nach einem Sturz im häuslichen Umfeld bei starken Schmerzen im rechten Bein bodengebunden über den Rettungsdienst in unserer Notaufnahme vorstellte. In der radiologischen Bildgebung zeigte sich eine rechtsseitige pertrochantäre Femurfraktur mit unfallchirurgischer Indikation zur operativen Versorgung mittels Femurnagel. Bei der anästhesiologischen präoperativen Evaluation konnten als relevante Vorerkrankungen ein arterieller Hypertonus, eine COPD sowie eine chronische Niereninsuffizienz eruiert werden. Die Patientin berichtete zudem von einer zunehmenden Vergesslichkeit in den letzten Monaten, welche am ehesten im Rahmen einer Demenz gewertet wurde. Die Eigenmedikation der Patientin umfasste Bromazepam 6–0–4,5 mg und Gabapentin 600-600-600 mg sowie Allopurinol, L‑Thyroxin, Ramipril, Torasemid, Bisoprolol jeweils p.o. und Salmeterol/Fluticason inhalativ.

## Therapie und Verlauf

Aufgrund der vorbestehenden COPD wurde bezüglich des anästhesiologischen Managements eine Spinalanästhesie mit Sedierung bevorzugt und entsprechend geplant. Die Einnahme der Eigenmedikation wurde bis zum Operationszeitpunkt fortgeführt (Torasemid, Bisoprolol) bzw. ab Krankenhausaufnahme pausiert (Bromazepam, Ramipril). Unmittelbar präoperativ zeigte sich die Patientin mit einem GCS von 15 Punkten sowie einem RASS („Richmond Agitation Sedation Scale“) von 0. Der Eingriff konnte am Folgetag der Krankenhausaufnahme problemlos durchgeführt werden. Die Spinalanästhesie erfolgte mittels spinaler Injektion von 4,2 ml Ropivacain 0,5 % sowie 7,5 µg Clonidin [1]. Zur Analgesie bei Lagerungsmaßnahmen erhielt die Patientin zweimalig 5 µg Sufentanil i.v. Die Sedierung erfolgte mittels kontinuierlicher i.v.-Infusion von Dexmedetomidin mit 20–30 µg/h in einer Gesamtdosis von knapp 50 µg. Postoperativ wurde die Patientin planmäßig in den Aufwachraum aufgenommen. Etwa 1 h postoperativ zeigte die Patientin eine deutlich reduzierte Vigilanz (RASS −3) ohne erneute Gabe eines Sedativums. Eine arterielle Blutgasanalyse zeigte eine deutliche respiratorische Azidose mit einem p_a_CO_2_ von 88,8 mm Hg und einem pH-Wert von 7,11. Bei der Verdachtsdiagnose einer Vigilanzminderung aufgrund einer Hyperkapnie wurde nach frustraner CPAP-Therapie eine notfallmäßige Narkoseeinleitung mittels Applikation von Sufentanil, Propofol und Rocuronium durchgeführt. Ein sofort im Anschluss durchgeführtes CT ergab keinen Hinweis auf ein akutes zerebrales Geschehen. Die Patientin wurde im Anschluss auf unsere interdisziplinäre Intensivstation übernommen. Am Folgetag zeigte sich ein akutes Nierenversagen (AKIN-Stadium 1). Nach Sedierungsende zeigte die Patientin im kurzfristigen Verlauf einen stark fluktuierenden neurologischen Zustand (RASS 0 bis −3) mit intermittierenden kurzen Wachheitsphasen im Wechsel mit Phasen ausgeprägter Vigilanzminderung. Da differentialdiagnostisch neben einem hypoaktiven Delir und einer postoperativen pulmonalen Komplikation auch der chronische Benzodiazepinkonsum als für die Vigilanzminderung ursächlich in Betracht kamen, erfolgte die probatorische, fraktionierte zweimalige i.v.-Gabe von 0,2 mg Flumazenil. Daraufhin zeigte sich eine zügige und deutliche Besserung des neurologischen Status der Patientin mit Wachheit, gebesserter Kontaktfähigkeit sowie gebesserter Aufforderungsmotorik. Die Patientin konnte dann problemlos extubiert werden. Mit Nachlassen der Flumazenilwirkung (Halbwertszeit ca. 40–80 min) kam es erneut zu einer Vigilanzminderung, welche nach der nochmaligen i.v.-Gabe von 0,2 mg Flumazenil erneut gebessert war. Aufgrund der angegebenen Konstellation begannen wir mit einer kontinuierlichen Flumazenilinfusion mit 0,2 mg/h. Wir verabreichten zudem eine antikonvulsive Entzugskrampfprophylaxe mittels Levetiracetam (500 mg 2‑mal täglich bei Niereninsuffizienz). Im Verlauf war eine Steigerung der kontinuierlichen Gabe von Flumazenil auf maximal 0,4 mg/h notwendig, um eine signifikante und nachhaltige Verbesserung der Kontaktfähigkeit und der Vigilanz zu erreichen. Nach 72-stündiger Flumazenilinfusion reduzierten wir die Rate auf 0,2 mg/h, woraufhin sich jedoch eine erneute und zügige Vigilanzminderung einstellte. Wir führten daher die Flumazenilinfusion mit 0,3 mg/h über weitere 24 h fort und reduzierten anschließend erneut auf 0,2 mg/h. Nach weiteren 24 h und somit in Summe 122,5 h konnten wir die Flumazenilinfusion am 8. postoperativen Tag (POD 8) schließlich beenden. Die Nierenretentionsparameter zeigten bereits kurz nach der Aufnahme auf die Intensivstation eine rückläufige Tendenz und besserten sich vor der Verlegung auf Normalwerte. Am 11. postoperativen Tag konnte die Patientin auf eine Überwachungsstation im Haus und am 13. postoperativen Tag auf die Normalstation verlegt werden. Die Patientin wurde vor der Verlegung entsprechend über die Notwendigkeit eines Entzugs informiert.

## Diagnostik

**Abb. 1:**
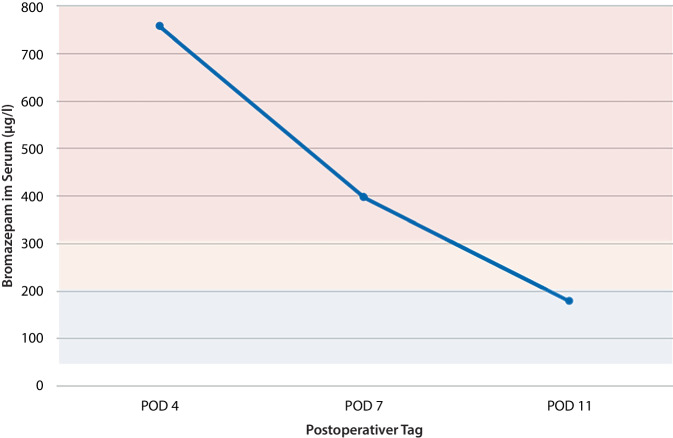
Verlauf der Bromazepamkonzentration im Serum in µg/l. *Blau hinterlegt*: therapeutischer Bereich 50–200 µg/l; *rot hinterlegt*: toxischer Bereich > 300 µg/l

Die Diagnosestellung einer akuten Benzodiazepinintoxikation erfolgte klinisch ex juvantibus. Zu Beginn der kontinuierlichen Flumazenilgabe über Perfusor am 4. postoperativen Tag erfolgte die erstmalige Bestimmung der Bromazepamkonzentration im Serum. Die Messung wurde am 7. und am 11. postoperativen Tag wiederholt und zeigte einen regelrechten Abfall im Verlauf. Der Zielspiegel von Bromazepam liegt bei 50–200 µg/l, wohingegen der toxische Bereich mit > 300 µg/l angegeben wird. Die gemessenen Werte zu den oben genannten Zeitpunkten lagen bei 756, 396 bzw. 178 µg/l und sind in Abb. 1 dargestellt.

Im Rahmen des berichteten intensivstationären Aufenthalts erfolgte keine Benzodiazepingabe.

## Diskussion

In dem dargestellten Fall handelt es sich um einen außergewöhnlichen postoperativen Verlauf nach initial komplikationsloser operativer Versorgung einer Femurfraktur unter Spinalanästhesie. Bei vorbekannter beginnender Demenz zeigte sich die Patientin präoperativ kontakt- und einwilligungsfähig. Postoperativ kam es zu einem Coma hypercapnicum mit respiratorischer Azidose, welche im intensivstationären Setting mit Flumazenil therapiert werden konnte [2, 3]. Die Arbeitshypothese war somit eine akute Benzodiazepinintoxikation bei chronischem Bromazepamabusus seit anamnestisch 30 bis 40 Jahren.

Bei der intraoperativen Verwendung von nur geringen Dosen an Sedativa ist die Vermutung der Autoren, dass sich der Pathomechanismus des dargestellten Falles durch die Verstoffwechselung des Bromazepams sowie die Umverteilung aus dem Fettgewebe bei langjähriger Einnahme eines Benzodiazepins mit einer Eliminationshalbwertszeit von 15–28 h und bei adipöser Patientin erklären lässt [4]. Bromazepam ist ein Substrat des Cytochrom-P450-Systems, insbesondere wird es über CYP1A2, CYP2D6 und/oder CYP2C19 verstoffwechselt [5–8]. Aus der Packungsbeilage für Dexmedetomidin geht hervor, dass der α_2_-Agonist womöglich eine Hemmung von CYP-Enzymen verursacht. In der Literatur wird Dexmedetomidin als Inhibitor von CYP2D6 und CYP2C19 genannt [9, 10]. Zudem lieferten In-vitro-Studien Hinweise, dass auch in vivo ein Interaktionspotenzial besteht zwischen Dexmedetomidin und Substraten, die überwiegend über CYP2B6 metabolisiert werden. Darüber hinaus handelt es sich bei Ramipril um einen Inhibitor von CYP2D6 und CYP3A4 sowie bei Torasemid um einen Inhibitor von CYP3A4 und CYP2C19 [10]. Beide Medikamente nahm die Patientin bereits über Jahre, jedoch haben auch diese Substanzen theoretisch das Potenzial, die Wirkung von Bromazepam zu erhöhen, insbesondere in einer Situation einer zusätzlichen Nierenfunktionseinschränkung. Es erscheint daher durchaus möglich, dass diese Konstellation in Kombination mit der akuten Erkrankung, dem akuten Nierenversagen sowie der Frailty der 74-jährigen Patientin dazu geführt haben, dass das fragile Gleichgewicht zwischen Bromazepamkonzentration im Blut und ausreichender, wenngleich reduzierter Vigilanz im Alltag nun perioperativ erheblich gestört wurde und so die subakute symptomatische Bromazepamintoxikation ausgelöst wurde. Somit ist eine erhöhte Wachsamkeit bei Patienten mit chronischem Benzodiazepinabusus seitens behandelnder Ärztinnen und Ärzte nötig, da diese Konstellation auch sekundär zu Vigilanzminderungen und damit einhergehenden Komplikationen führen kann.

## Fazit für die Praxis

Bei Patienten mit langjährigem Benzodiazepinabusus ist besondere Vorsicht geboten, da es in dieser Konstellation u. a. auch trotz niedriger Sedativadosierung und nach initial guter Vigilanz zu einer sekundären neurologischen Verschlechterung kommen kann.
